# Strategic honey bee hive placement improves honey bee visitation but not pollination in northern highbush blueberry

**DOI:** 10.1093/jee/toae267

**Published:** 2024-11-09

**Authors:** Kayla Brouwer, Maxime Eeraerts, Emma Rogers, Lauren Goldstein, Jacquelyn A Perkins, Meghan O Milbrath, Andony Melathopoulos, Jason Meyer, Clark Kogan, Rufus Isaacs, Lisa Wasko DeVetter

**Affiliations:** Department of Horticulture, Washington State University, Northwestern Washington Research and Extension Center, Mount Vernon, WA, USA; Department of Horticulture, Washington State University, Northwestern Washington Research and Extension Center, Mount Vernon, WA, USA; Department of Environment, Forest and Nature Lab, Ghent University, Melle-Gontrode, Belgium; Department of Horticulture, Washington State University, Northwestern Washington Research and Extension Center, Mount Vernon, WA, USA; Department of Entomology, Michigan State University, East Lansing, MI, USA; Department of Entomology, Michigan State University, East Lansing, MI, USA; Department of Entomology, Michigan State University, East Lansing, MI, USA; Department of Horticulture, Oregon State University, Corvallis, OR, USA; Peerbolt Crop Management, Portland, OR, USA; StatsCraft LLC, Spokane, WA, USA; Department of Entomology, Michigan State University, East Lansing, MI, USA; Department of Horticulture, Washington State University, Northwestern Washington Research and Extension Center, Mount Vernon, WA, USA

**Keywords:** *Apis mellifera*, bees, crop pollination, hive placement, *Vaccinium*

## Abstract

Commercial blueberry *Vaccinium* spp. (Ericales: Ericaceae) production relies on insect-mediated pollination. Pollination is mostly provided by rented honey bees, *Apis mellifera* L. (Hymenoptera: Apidae), but blueberry crop yields can be limited due to pollination deficits. Various hive placement strategies have been recommended to mitigate pollination shortfalls, but the effect of hive placement has received limited formal investigation. This study explores the effects of clumped and dispersed hive placement strategies on honey bee visitation and pollination outcomes in “Bluecrop” and “Duke” fields over 2 years (2021 and 2022) within 2 economically important regions of production in the United States—the Midwest (Michigan) and Pacific Northwest (Oregon and Washington). Clumping hives consistently increased honey bee visitation rate but did not result in higher fruit set, fruit weight, or seed count. Increases in honey bee visitation through clumping could perhaps improve pollination outcomes in more pollination-limited blueberry cultivars and other pollination-dependent crops. Clumping hives is substantially more efficient and cost-effective for beekeepers due to fewer drop locations and could lead to cost savings for both beekeepers and blueberry growers without growers sacrificing pollination levels and crop yields.

## Introduction

Production of blueberry, *Vaccinium* spp. (Ericales: Ericaceae), has undergone rapid expansion worldwide within the past 2 decades (Brazelton et al. 2021, Eeraerts et al. 2023a). This crop has the potential to achieve nearly 100% fruit set but is highly dependent on the quality of pollination and field management (Ehlenfeldt 2001, Arrington and DeVetter 2018, Kumarihami et al. 2021). Ensuring sufficient pollination in commercial blueberry fields is challenging, as pollen limitation has been frequently observed, restricting fruit set, berry weight, and yield potential (Reilly et al. 2020, Eeraerts et al. 2024). More specifically, pollen limitation and the contribution of honey bees and wild bees to blueberry pollination have been reported to vary among different cultivars and production regions, which has downstream impacts on yields (Kendall et al. 2020, Cortés-Rivas et al. 2023, Eeraerts et al. 2023a, 2024, Ramírez-Meíja et al. 2023).

Flowers of blueberry are not readily self-pollinated through passive means as the anthers release pollen through poricidal dehiscence, which benefits from buzz pollination, a type of pollination in which bees use sonication to release pollen (Buchmann 1983). Honey bees, *Apis mellifera* L. (Hymenoptera: Apidae), are the most common pollinator visiting commercial blueberry fields (Eeraerts et al. 2023a), but they are not capable of buzz pollination and only incidentally pollinate blueberry by brushing against anthers while foraging primarily for nectar (Dogterom and Winston 1999, Hoffman et al. 2018). Bumblebees *Bombus* spp. (Hymenoptera: Apidae) and some solitary bee species can buzz pollinate and are thus comparatively more efficient pollinators of blueberry on a single-visit basis (Rogers et al. 2013, Cardinal et al. 2018, Eeraerts et al. 2023a). Yet, native populations are generally too low in abundance for commercial growers to rely on them solely for pollination services in modern, large-scale blueberry fields (Isaacs and Kirk 2010, Benjamin and Winfree 2014, Gibbs et al. 2016, Eeraerts et al. 2023b). Blueberry growers consequently depend on rented honey bee hives to provide pollination services but are seeking strategies to improve honey bee mediated pollination in this difficult-to-pollinate crop (Estravis-Barcala et al. 2021, Mallinger et al. 2021, DeVetter et al. 2022, Eeraerts et al. 2023b).

**Fig. 1.**
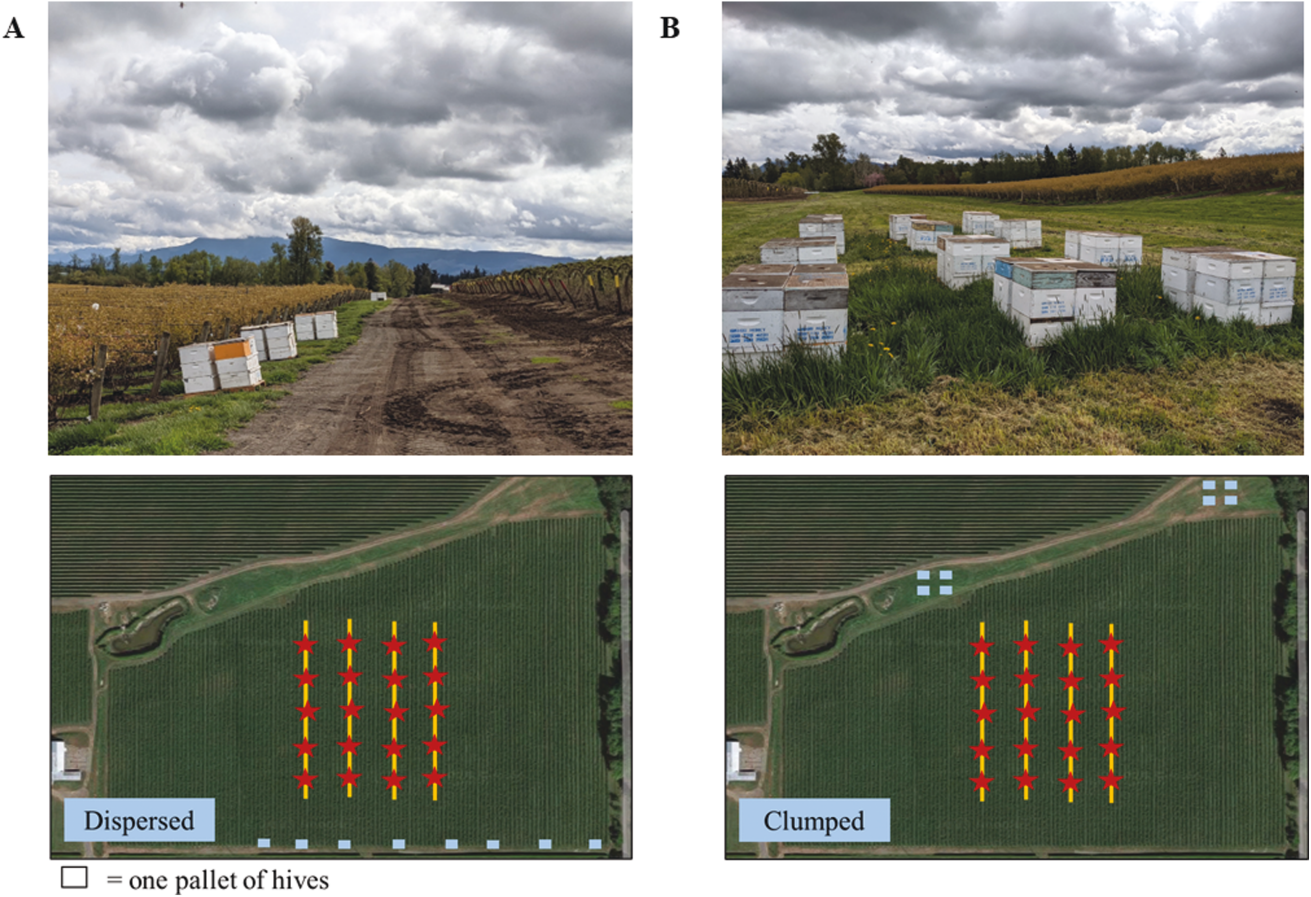
Honey bee hive placement strategies commonly employed in commercial blueberry fields include dispersed (A) or clumped (B) configurations. The dispersed configuration is characterized by single pallets of hives spread along the field edge(s), whereas the clumped configuration is characterized by several pallets of hives concentrated in drop locations away from the field edge. Schematic diagrams below the photos depict hive placement as well as the location of the 4, 100-m transects (yellow lines) and observational bushes (red stars). Photos by L.W. DeVetter.

Hive placement has the potential to influence pollination outcomes, but this has undergone little formal evaluation. Many growers request beekeepers disperse hives adjacent to and along field edges with the assumption that hive proximity will increase and encourage more even foraging throughout the field (Fig. 1). Modeling efforts using the dioecious crop kiwi *Actinidia chinensis* Planch. (Ericales: Actinidiaceae) support this approach and indicate fruit set will be greater when hives are distributed evenly around a field relative to hives located in a single point (Li et al. 2022). Yet, empirical data are lacking. One field study found a “precision-management” approach with hives distributed around the entirety of a field increased pollination success and yields of blueberry, but hives in the precision-management treatment were carefully preselected for quality before field deployment and provided intensive care that included a dietary supplement and coordinated pesticide applications whereas the control did not (Cavigliasso et al. 2021). Therefore, the effects of hive placement in this study may be confounded with differences in apicultural practices. From a beekeeper’s perspective, concentrating hives in fewer drop locations via clumping is considerably more efficient than dispersing hives around the field, particularly in regions where the demand for honey bee hives is large and overlaps with other fields and crops. It is therefore essential to understand the implications of hive placement through field-level evaluations to ensure sufficient crop pollination while minimizing unnecessary labor, time, and fuel costs for pollinating beekeepers.

Blueberry growers and beekeepers need strategies to optimize honey bee pollination while protecting bees from pesticide exposure, and hive placement may be 1 component of integrated crop pollination plans that can support the goals of both groups (Isaacs et al. 2017, DeVetter et al. 2022). In this study, 2 hive placement strategies commonly employed in commercial northern highbush blueberry *Vaccinium corymbosum* L. (Ericales: Ericaceae) systems were compared across multiple growing regions with the hive placement strategies identified as “clumped” and “dispersed” (Fig. 1; *see description in the methods*). Specific research questions addressed in this study include the following: (i) How does honey bee hive placement affect honey bee visitation on northern highbush blueberry crop flowers? and (ii) How does honey bee hive placement affect fruit set, berry weight, and seed count in northern highbush blueberry? Fields in the Midwest (Michigan) and Pacific Northwest (Oregon and Washington) were included in the study. Together, these production regions collectively represent 47% of the total area of production and ≈55% of the supply of cultivated blueberry in the United States (USDA NASS 2024). Results from this study conducted across multiple production regions provide critical insights to inform honey bee placement strategies in northern highbush blueberry, with application to other pollinator-dependent crops that utilize honey bees.

## Materials and Methods

### Study Overview

Data were collected in 2021 and 2022 in blueberry fields in the Midwest (Michigan) and Pacific Northwest (Oregon and Washington; Supplementary Fig. S1). Eight sites were utilized in the Midwest in 2021 and 2022. In the Pacific Northwest, data from Oregon were collected from 6 fields in 2021 and 5 fields in 2022, and Washington data were collected from 12 sites in both 2021 and 2022 (*n* = 26 and 25 total sites in 2021 and 2022, respectively; Supplementary Table S1). Sites were selected based on several criteria. Cultivar was standardized within each region, and all fields were established (6 years or older) and managed using conventional commercial production practices. “Bluecrop” was studied in the Midwest while “Duke” was the focal cultivar in the Pacific Northwest. To maintain independence, field sites were a minimum of 2 km away from other experimental sites, and sites were selected to ensure landscape was not correlated with treatment. Fields with nearby apiaries or crops with overlapping blooms were excluded, and beekeeper variation within a state was kept to a minimum. An effort was made to keep hive stocking density consistent within a region, but there was some variation in reported and actual hive density. Actual field-level honey bee hive densities ranging between 1.36 and 9.35 hives per hectare (4.23 ± 1.87 and 4.01 ± 1.71 hives per hectare in 2021 and 2022, respectively (mean ± SD) and see Supplementary Table S1). The honey bee stocking density for each region varied to reflect local pollination practices but was not correlated with treatment (Supplementary Table S2; Supplementary Fig. S2).

For each state and year, approximately half of the fields were assigned the dispersed treatment and half to the clumped treatment (Supplementary Table S1). Hive placement treatments were consistent for the same fields in the 2 years of sampling, except for 1 farm in Oregon and 1 in Washington. Treatment assignments were not completely random, as some farms did not have the space for the clumped treatment (Supplementary Table S1). The dispersed treatment was characterized by single pallets of hives spread along the field edge, whereas the clumped treatment was characterized by several pallets of hives concentrated in 1 to a few drop locations around the field (Fig. 1). Each pallet in Michigan and Washington contained 4 hives, while there were 6 hives per pallet in Oregon. Hives were placed so they were in proximity of the blueberry field; however, distance between the hives and field edges was not consistently measured across all sites and is thus not reported. Hives in the dispersed treatment were on average at least 30 cm away from the field edge, while the distance of the hives in the clumped treatment varied from 6.0 to 17 m away from the field edge, with 1 field in Washington with hives 508 m away from the field edge. Field size ranged from 1.38 to 8.39 ha (5.04 ± 2.33).

### Pollinator Visitation Assessments

Honey bee and other wild insect pollinator visitation was measured using a modified version of the scan sampling protocol (Vaissière et al. 2011). In each of the study sites, 4, 100-m-long transects were established prior to data collection. Transects ran along the length of a row and were in a fixed position near the geometric center of the field with at least 5 m between each transect. This approach permitted estimation of the average pollinator density within a field. In 2022, transects were established on alternating sides of the row to avoid fruit damage due to sunburn from intense heat and ultraviolet radiation exposure which occurred in the Pacific Northwest during an extreme heat event that occurred in the summer of 2021. Transects started at least 9 m from field edges to avoid potential edge effects. Scan sampling was conducted once during mid-bloom at each field between 1,000 and 1,600 h by walking the length of each transect slowly. While walking the transect, the number of honey bees and wild bees visiting open blueberry flowers was recorded (wild bee data not presented). Due to the size of bushes and inability to confidently count pollinators throughout the whole bush, estimates of pollinator visitation only occurred on the half of the bush facing the observer. Each transect walk was timed and took approximately 10 min. Weather conditions (air temperature, solar radiation, cloud cover, humidity, and barometric pressure) were recorded before and after each scan sampling event. At each site, scan sampling was performed during mid-bloom and during optimal weather for honey bees, which is sunny-to-partly sunny, air temperatures above 13 °C, and low wind speeds (Vaissière et al. 2011).

### Pollination Outcomes

Within each transect, pollination outcomes, including fruit set, berry weight, and seed count, were measured on 5 focal bushes that were representative of the field and distributed along the transect (*n* = 20 bushes per field). Each bush had one branch at mid-canopy height that was labeled with flagging tape before bloom and had flowers counted twice during the bloom period for fruit set determination. After bloom, focal branches were covered with fine mesh bags to protect developing berries from bird depredation, insect damage, and accidental harvest. Prior to commercial harvest, ripe, blue fruits were collected, weighed, and counted to determine fruit set. If multiple harvests occurred, collected berries were maintained in cold storage and pooled across harvests by field. By means of the total berry count and total weight, we determined the weight per 100 berries (gram) as a measure of yield. After harvest, 20 pooled berries per field were randomly selected to determine the number of seeds that were extracted and enumerated. Only seeds that were large, dark brown, and considered viable were enumerated (see Eeraerts et al. 2023b for methods).

### Statistical Analyses

To determine the effect of hive placement treatment (i.e., clumped vs. dispersed) on honey bee visitation, we used a linear mixed-effects model through R version 4.1.1 (LME, function *lme*, R package *nlme;*Pinheiro and Bates 2019; and R Core Team 2021). In this model, honey bee visitation was considered a response variable and hive placement treatment, honey bee hive density, region, and their interaction were included as fixed variables (full model: *y* = Region*HivePlacement + Region*HiveDensity). While the main focus of the study was on hive placement, hive density was included as a possible explanatory variable, and correlation analysis showed there were no correlations between hive placement and field-level stocking densities across all locations and years (Supplementary Table S2; variance inflation factors were also calculated and were <2 for all factors). Two random effects, sampling year nested in Field ID, were included as random variables to account for correlated errors within field sites. Honey bee visitation was calculated as honey bees/transect/hour. For the second question, we used LME models to determine the effect of hive placement treatment on the following response variables: (i) blueberry fruit set, (ii) berry weight per 100 berries, and (iii) seed count. In these models, hive placement treatment, hive density, region, and their interactions were included as fixed variables, and sampling year nested in Field ID were included as random variables.

Before running each model, we checked the response for normality and outliers. For each question and response variable, we tested and reported the full model. After running the models, model fit of the LME model was evaluated visually by checking the normality of the model residuals (QQ-plot and plot of the residuals vs. the fitted values). For honey bee visitation, we applied a log transformation in order to normalize this response variable and its residuals.

## Results

### Pollinator Visitation

**Fig. 2.**
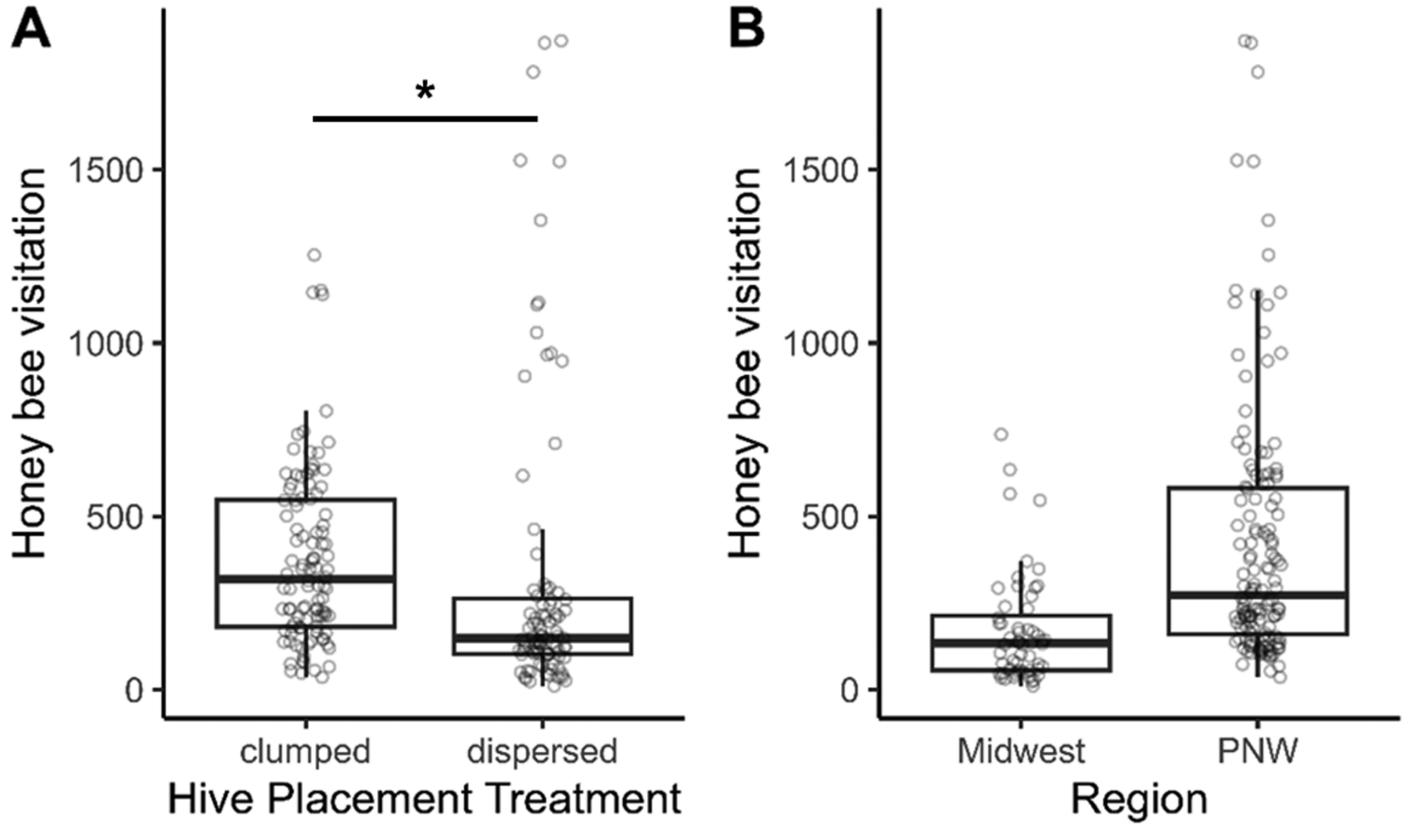
Honey bee visitation (expressed as a rate of honey bee number per 100-m transect per hour) as a function of the different honey bee hive placement treatments (A) and study regions (B), 2021–2022. Regions include the Midwest (Michigan) and Pacific Northwest (PNW; Oregon and Washington). Asterisk denotes significant differences (*P* < 0.05).

In total, we counted 34,692 honey bee visits, 965 wild bee visits, and 557 other wild insect visits to blueberry blossoms. We found that fields with clumped hives had greater honey bee visitation compared to fields with dispersed hives (Fig. 2A; Tables 1 and 2). Honey bee hive density and region did not affect honey bee visitation, and there was no significant interaction between region and hive placement and region and hive density (Fig. 2B; Tables 1 and 2). In the Midwest, it was estimated that clumping hives resulted in a 2.6-fold increase [95% CI (0.9, 7.1)] in the average number of honey bees visiting blueberry flowers. In the Pacific Northwest, the increase was 1.3-fold [95% CI (0.7, 2.4)]. Honey bee visitation was weakly correlated with temperature during sampling (Supplementary Table S3).

**Table 1.** Linear mixed-effect models assessing the effects of honey bee hive placement, honey bee hive density, and region on honey bee visitation, fruit set, berry weight, and seed count in northern highbush blueberry. Full models are reported with their marginal *R*², *F*-statistic, and *P*-values

Response variable	Full model	*R*² m	Fixed variables	*F*	*P*
Honey bee visitation	Region*Placement + Region*Density	0.31	Region	2.68	0.11
			**Placement**	**4.77**	**0.04**
			Density	0.12	0.73
			Region:Placement	1.51	0.24
			Region:Density	0.17	0.69
Fruit set	Region*Placement + Region*Density	0.10	Region	0.65	0.43
			Placement	0.00	0.99
			Density	1.56	0.23
			Region:Placement	0.73	0.40
			Region:Density	0.67	0.42
Weight per 100 berries	Region*Placement + Region*Density	0.01	Region	0.19	0.67
			Placement	0.28	0.60
			Density	0.01	0.91
			Region:Placement	0.73	0.40
			Region:Density	0.02	0.89
Seed count per berry	Region*Placement + Region* Density	0.09	Region	0.52	0.48
			Placement	0.56	0.46
			Density	0.30	0.59
			Region:Placement	1.72	0.21
			Region:Density	2.49	0.13

**Table 2.** Mean and standard errors (SE) of honey bee visitation, fruit set, berry weight, and seed count in northern highbush blueberry for each level of honey bee hive placement and region [Midwest (Michigan) and Pacific Northwest (PNW; Oregon and Washington)], 2021–2022. Presented values of honey bee visitation represent back-transformed (non-log transformed) data

Variable	Treatment	Treatment level	Mean	SE
Honey bee visitation	Placement	Clumped	377	24.8
		Dispersed	322	45.6
	Region	Midwest	170	21.1
		PNW	424	32.2
Fruit set	Placement	Clumped	85.7	0.81
		Dispersed	85.5	0.86
	Region	Midwest	77.3	1.19
		PNW	89.5	0.60
Weight per 100 berries	Placement	Clumped	149.0	1.93
		Dispersed	147.0	1.98
	Region	Midwest	153.0	2.27
		PNW	145.0	1.73
Seed count per berry	Placement	Clumped	23.9	0.66
		Dispersed	21.9	0.68
	Region	Midwest	18.6	0.72
		PNW	25.3	0.60

### Pollination Outcomes

**Fig. 3.**
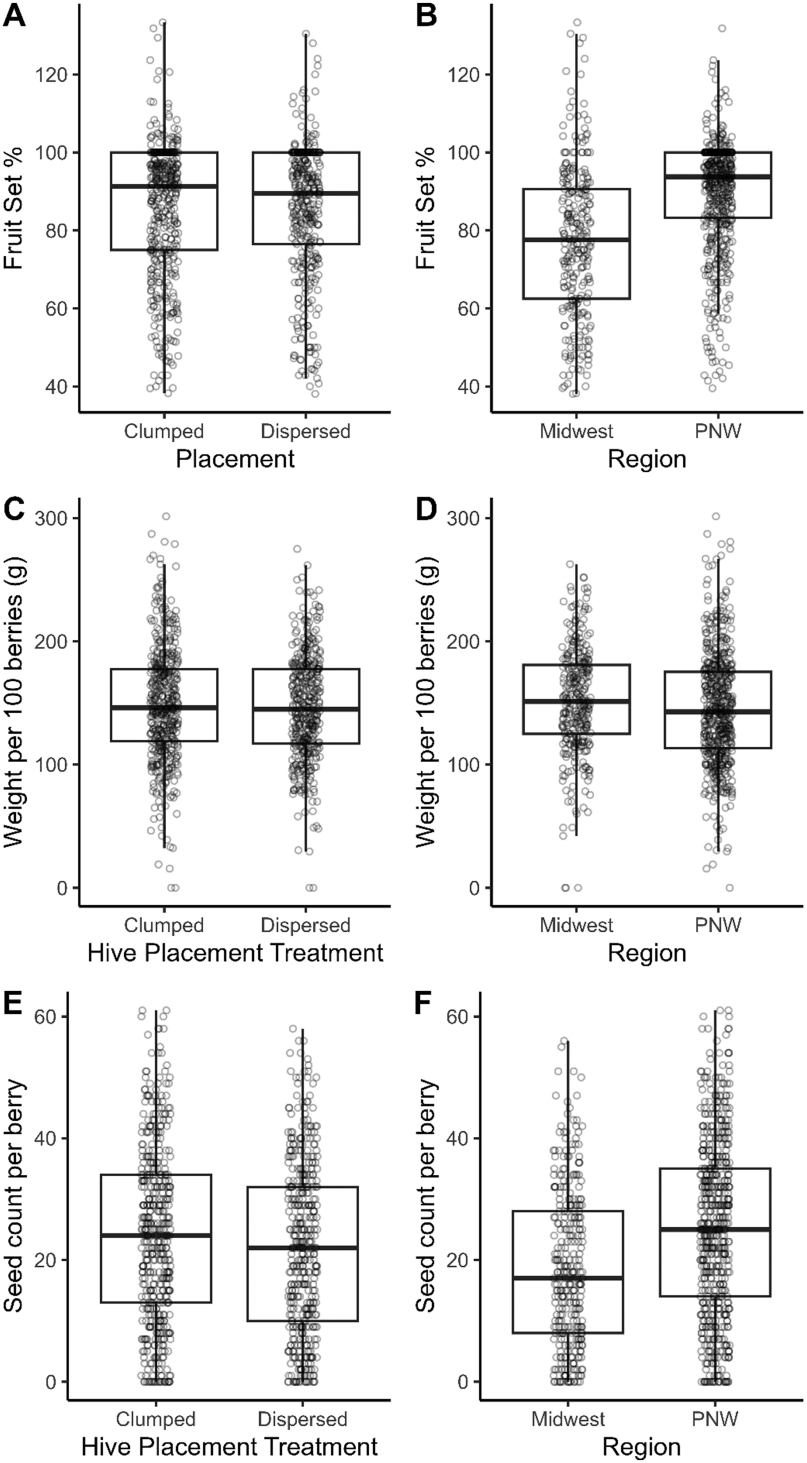
Pollination outcomes of highbush blueberry expressed as fruit set (%, A, B), berry weight per 100 berries (C, D), and seed count per berry (E, F) as a function of hive placement treatment and study region in the Midwest (Michigan) and Pacific Northwest (PNW; Oregon and Washington), respectively. Data were collected between 2021 and 2022.

Region, hive placement, hive density, and their interactions did not influence blueberry fruit set, weight per 100 berries, or seed count per berry (Tables 1 and 2; Fig. 3). Outliers were detected for both fruit set (*n* = 93) and weight per 100 berries (*n* = 33), yet excluding these outliers gave the same results (Supplementary Table S4).

## Discussion

To our knowledge, this is the first robust study that empirically evaluated how honey bee hive placement strategies influence honey bee visitation and pollination outcomes in a pollinator-dependent crop. Results generated from data collected over 2 years and 2 production regions showed clumping hives increases honey bee visitation. In addition, pollination outcomes that can serve as indicators of yield were not affected by hive placement, nor did hive density or region affect these variables. This is interesting as regional variation in honey bee contributions to blueberry and apple *Malus domestica* Borkh. (Rosales: Rosaceae) pollination has been previously documented (Gibbs et al. 2016, Garratt et al. 2021, Eeraerts et al. 2023a). Furthermore, cultivar differed by region, and it is known that there is variation in pollen limitation, the extent that bees can overcome pollen limitation, and the relationship between seed number and berry weight across blueberry cultivars (Strik and Vance 2019, Kendall et al. 2020, Cortés-Rivas et al. 2023, Eeraerts et al. 2024). Honey bees also have different preferences for blueberry floral morphologies, and this can translate into different visitation rates (Courcelles et al. 2013).

The ability of hive placement strategies to influence honey bee visitation is not well characterized in the literature. Furthermore, it is not clear how clumping the colonies increases floral visitation in this study. Additional research on other related variables, including honey bee behavior, colony strength, and pesticide drift, may lead to greater understanding of the underlying mechanism(s). Nevertheless, this work has direct implications for beekeepers and associated pollination costs. When hives are spread throughout the field, it results in extra time, fuel, and labor for beekeepers during placement, colony management visits, and hive removal. The results of this project indicate that the costly practice of spreading hives along field edges is unnecessary and does not improve crop pollination.

Explanations regarding the mechanisms driving the positive effects of clumping on honey bee visitation are speculative. Nonetheless, we put forward some potential explanations while emphasizing that this topic requires more empirical research. One possibility is that clumping may result in increased communication between hives due to the close proximity of many colonies in space. Honey bees rely on highly specialized dances and pheromone cues to communicate the availability and location of floral resources and recruit other foragers to these locations (von Frisch 1993, Dornhaus and Chittka 2004, Bortolotti and Costa 2014). Although honey bees are skilled at finding the location of their colonies, honey bee drift occurs when a forager enters the wrong hive, and this can occur frequently when hives are close together, face the same direction, and/or are the same color (Free 1958, Boylan-Pett and Hoopingarner 1991, Šekulja et al. 2014). The proximity of hives in the clumped treatment may lead to more instances of drift that increases communication between hives, resulting in increased foraging fidelity to blueberry blossoms. Clumping could also increase honey bee visitation in fields by limiting the availability of floral resources close to the colonies. Honey bees prefer to forage near their colonies when floral resources are available (Steffan-Dewenter and Kuhn 2003, Pascual Tudanca et al. 2024). When hives are clumped, nearby floral resources may be more quickly depleted, which could manifest into more foragers looking for floral resources further away from the colony and adjacent crops.

Although clumping hives resulted in greater honey bee visitation, the hive placement treatment did not affect pollination of blueberry under the conditions of the study. Pollination may have been sufficient regardless of differences in honey bee visitation (Gibbs et al. 2016, Eeraerts et al. 2023b, 2024). Individual blueberry flowers require 30–100 pollen tetrads to maximize fruit set and berry size, although this number has been found to depend on whether the pollen is self or outcrossed (Dogterom et al. 2000, Sun et al. 2021). However, more recent work with southern highbush blueberry suggests optimal pollen deposition ranges between 112 and 274 tetrads (Ramírez-Mejía et al. 2024b, and reviewed in DeVetter et al. 2022). Blueberry pollen tetrad load varies by body subregion on a honey bee and ranges from 16 tetrads on the fore tarsal claws to 400 tetrads on the basitarsi (Hoffman et al. 2018). This indicates that a single floral visit by a honey bee could lead to sufficient pollen transfer to completely pollinate the flower, although other studies report 5–15 honey bee visits are required to optimize stigmatic pollen deposition or maximize blueberry fruit weight (Danka et al. 1993, Rogers et al. 2013, Kendall et al. 2020, Ramírez-Mejía et al. 2024b). Additionally, pollination limitations can be cultivar-specific and influence insect pollination-dependency within a species (Guerra and Rodrigo 2015, Garratt et al. 2021). Indeed, Eeraerts et al. (2024) synthesized pollination deficits in northern highbush blueberry across 11 studies and showed “Duke” experienced no pollination deficits, whereas “Bluecrop” experienced deficits in berry weight and seed count. “Duke” is one of the most widely planted blueberry cultivar in the Pacific Northwest (Strik et al. 2014), which is why it was selected for the study. However, these findings emphasize the importance of considering cultivars with varying pollination dependencies to better determine the impacts of pollination treatments on yield components (Kendall et al. 2020, Cortés-Rivas et al. 2023, Ramírez-Mejía et al. 2023, Eeraerts et al. 2024). Many management factors also influence pollination outcomes in blueberry. Field age, bush density, weed management style, irrigation, use of mulches, soil conditions, fertilizer use, pest and disease management, and use of plant growth regulators all contribute to blueberry yields and can interact with each other and influence the pollination success of blueberry and other crops (Melathopoulos et al. 2014, Retamales et al. 2015, Hanson and Kelsey 2019, Tamburini et al. 2019). Isolating a single factor to evaluate its effects on insect-mediated pollination is challenging and more controlled studies are needed to untangle the influence of these factors.

Placement of hives close together increased honey bee visitation, which has the potential to increase yield outcomes in blueberry cultivars that are pollination limited as well as other pollinator-dependent crops. Studies on the effect of hive placement on honey bee visitation and berry outcomes are limited. A modeling study in apple assessed the impact of hive spatial arrangements on pollination and found distance between hives to be unimportant, whereas hive density, proportions of focal crop, natural habitats, mass-flowering crops, and edge density in the landscape were significant predictors of floral visitation and pollination (Santibañez et al. 2022). Another study in almond *Prunus dulcis* (Mill.) DA Webb (Rosales: Rosaceae) showed spatial arrangement of hives affected pollination with fewer colonies per placement (<100) arranged so that more trees are within 400 m of colonies as being beneficial for pollination (Cunningham et al. 2016).

The lack of hive density effects on both honey bee visitation and blueberry pollination is interesting. Attempts were made at the onset of this study to standardize hive density across treatments, yet there were discrepancies in reported and actual hive densities that led to large variations in hive densities across sites and regions. The lack of an effect from variations in field-level hive density on honey bee visitation and pollination corresponds with other studies in blueberry (Benjamin and Winfree 2014, de Groot et al. 2015, Mallinger et al. 2021, Eeraerts et al. 2023b) and other crops including pumpkin *Cucurbita* spp. (Cucurbitales: Cucurbitaceae), apple, and almond (Peterson et al. 2013, de Groot et al. 2015, Alomar et al. 2018). Yet, sometimes positive effects of increasing stocking density are detected (blueberry: Benjamin and Winfree 2014, Arrington and DeVetter 2018, pumpkin: Artz et al. 2011, apple: Osterman et al. 2021, sweet cherry *Prunus avium* L. (Rosales: Rosaceae): Osterman et al. 2023). Variation across studies might be explained by the discrepancies in grower-reported and actual hive stocking densities (Benjamin and Winfree 2014). Additionally, large variations in hive densities in neighboring fields (Anders et al. 2023, Eeraerts et al. 2023b), variation in colony strength (Cavigliasso et al. 2021, Grant et al. 2021), or other aspects of the landscape surrounding the farm may contribute to these discrepancies similar to other mass-flowering crops or natural habitat (Eeraerts et al. 2017, Osterman et al. 2021). Indeed, the foraging range of honey bees extends far beyond field edges in which hives are placed (Visscher and Seeley 1982, Beekman and Ratnieks 2000, Steffan-Dewenter and Kuhn 2003); hence, we argue that a more holistic landscape perspective is required to optimize honey bee management at the farm level.

Hive strength or population size of the colony is another important variable rarely accounted for in pollination studies, with stronger hives associated with higher blueberry yields (Grant et al. 2021). Recent work has also demonstrated the intuitive interplay between hive quality and stocking density, with lower stocking densities being required to achieve maximum pollination in blueberry when hives are of high quality, whereas higher stocking densities are necessary to maximize pollination when hive quality is low (Ramírez-Mejía et al. 2024a). Colony strength was not controlled for in this study and represents one limitation given it is possible hives of variable quality were not randomly distributed across the experimental sites. Practically, however, the findings from this study combined with previous research highlight the relationships between stocking density, hive strength, and placement in the landscape, and these should all be factors considered when developing honey bee-encompassing pollination plans.

Overall, this study showed that honey bee hive placement should be considered within integrated crop pollination plans (Isaacs et al. 2017, DeVetter et al. 2022), with clumping hives increasing honey bee visitation compared to dispersed placement strategies within commercial blueberry fields. The mechanisms for increased honey bee visitation via clumping hives are speculatory at this time, and further research is needed to identify what drives increases in honey bee visitation. Although gains in honey bee visitation were not associated with improved pollination outcomes, it is important to underscore that declines in pollination were not observed with clumping, so there is no revenue reduction for the grower expected from this approach. Additionally, cultivars with greater pollination-dependency may in fact show improvements in pollination outcomes with increased honey bee visitation from clumped hives. The clumped configuration also has potential benefits that we will be exploring in ongoing and future studies, such as decreased exposure to pesticide drift and lower beekeeper costs for deploying, maintaining, and removing hives, given that this placement strategy entails fewer drops per field location. Hive placement is dictated by spatial considerations, and some fields may not have the area to select one placement strategy over another. Regardless, beekeepers, growers, and consultants can consider clumping hives when feasible, and this strategy is expected to increase honey bee visitation and potentially pollination outcomes in pollen-limited cultivars or crops. Greater honey bee visitation from clumping hives may also allow growers to reduce honey bee hive densities, which could lead to cost savings and mitigate concerns about resource competition with wild pollinators (Mallinger et al. 2017). Beekeepers also have the potential to improve their profitability through this more efficient placement strategy when field conditions permit.

## Supplementary data

Supplementary data are available at *Journal of Economic Entomology* online.

toae267_suppl_Supplementary_Figures_S1-S2_Tables_S1-S4

## Data Availability

The code of this study are available at 10.6084/m9.figshare.27179868, the data will be made public on figshare as well upon acceptance of the manuscript.

## References

[CIT0001] Alomar D , González-Estévez MA, Traveset A, et al 2018. The intertwined effects of natural vegetation, local flower community, and pollinator diversity on the production of almond trees. Agric. Ecosyst. Environ. 264:34–43. https://doi.org/10.1016/j.agee.2018.05.004

[CIT0002] Anders M , Grass I, Linden VMG, et al 2023. Smart orchard design improves crop pollination. J. Appl. Ecol. 60:624–637. https://doi.org/10.1111/1365-2664.14363

[CIT0003] Arrington M , DeVetter LW. 2018. Increasing honey bee hive densities promotes pollination and yield components of highbush blueberry in western Washington. HortScience 53:191–194. https://doi.org/10.21273/hortsci12644-17

[CIT0004] Artz DR , Hsu CL, Nault BA. 2011. Influence of honey bee, *Apis mellifera*, hives and field size on foraging activity of native bee species in pumpkin fields. Environ. Entomol. 40:1144–1158. https://doi.org/10.1603/EN10218 22251726

[CIT0005] Beekman M , Ratnieks FLW. 2000. Long‐range foraging by the honey‐bee, *Apis mellifera* L. Funct. Ecol. 14:490–496. https://doi.org/10.1046/j.1365-2435.2000.00443.x

[CIT0006] Benjamin FE , Winfree R. 2014. Lack of pollinators limits fruit production in commercial blueberry (*Vaccinium corymbosum*). Environ. Entomol. 43:1574–1583. https://doi.org/10.1603/EN13314 25313694

[CIT0007] Bortolotti, L, Costa C. 2014. Chemical communication in the honey bee society. In: Mucignat-Caretta C., editor. Neurobiology of chemical communication. Boca Raton (FL): CRC Press/Taylor & Francis. Available from: https://www.ncbi.nlm.nih.gov/books/NBK200983/. 24830041

[CIT0008] Boylan-Pett W , Hoopingarner R. 1991. Drifting of honey bee foragers within and between apiaries pollinating blueberry, *Vaccinium corymbosum*. Acta Hortic. 288:111–115. https://doi.org/10.17660/actahortic.1991.288.12

[CIT0009] Brazelton C , Fain C, Ogg M, et al Global state of the blueberry industry report-2021. International Blueberry Organization; 2021. p. 1–167. Available from: https://www.internationalblueberry.org/2022-report/.

[CIT0010] Buchmann, SL. 1983. Buzz pollination in angiosperms. In: Jones CE., Little RJ., editors Handbook of experimental pollination biology. New York: Scientific and Academic Editions; p. 73–113.

[CIT0011] Cardinal S , Buchmann SL, Russell AL. 2018. The evolution of floral sonication, a pollen foraging behavior used by bees (Anthophila). Evolution 72:590–600. https://doi.org/10.1111/evo.13446 29392714 PMC5873439

[CIT0012] Cavigliasso P , Negri P, Viel M, et al 2021. Precision management of pollination services to blueberry crops. Sci. Rep. 11:20453. https://doi.org/10.1038/s41598-021-00068-1 34650072 PMC8516932

[CIT0013] Cortés-Rivas B , Monzón VH, Rego JO, et al 2023. Pollination by native bees achieves high fruit quantity and quality of highbush blueberry: a sustainable alternative to managed pollinators. Front. Sustain. Food Syst. 7:1142623. https://doi.org/10.3389/fsufs.2023.1142623

[CIT0014] Courcelles DMM , Button L, Elle E. 2013. Bee visit rates vary with floral morphology among highbush blueberry cultivars (*Vaccinium corymbosum* L.). J. Appl. Entomol. 137:693–701. https://doi.org/10.1111/jen.12059

[CIT0015] Cunningham SA , Fournier A, Neave MJ, et al 2016. Improving spatial arrangement of honeybee colonies to avoid pollination shortfall and depressed fruit set. J. Appl. Ecol. 53:350–359. https://doi.org/10.1111/1365-2664.12573

[CIT0016] Danka RG , Lang GA, Gupton CL. 1993. Honey bee (Hymenoptera: Apidae) visits and pollen source effects on fruiting of ‘Gulfcoast’ southern highbush blueberry. J. Econ. Entomol. 86:131–136. https://doi.org/10.1093/jee/86.1.131

[CIT0017] de Groot A , van Kats RJM, Reemer M, et al 2015. De bijdrage van (wilde) bestuivers aan de opbrengst van appels en blauwe bessen: Kwantificering van Ecosysteemdiensten in Nederland. 41. Retrieved from: www.wageningenUR.nl/alterra.%0Ahttp://edepot.wur.nl/353774.

[CIT0018] DeVetter LW , Chabert S, Milbrath MO, et al 2022. Toward evidence-based decision support systems to optimize pollination and yields in highbush blueberry. Front. Sustain. Food Syst. 6:1006201. https://doi.org/10.3389/fsufs.2022.1006201

[CIT0019] Dogterom MH , Winston ML. 1999. Pollen storage and foraging by honey bees (Hymenoptera: Apidae) in highbush blueberries (Ericaceae), cultivar Bluecrop. Can. Entomol. 131:757–768. https://doi.org/10.4039/ent131757-6

[CIT0020] Dogterom MH , Winston ML, Mukai A. 2000. Effect of pollen load size and source (self, outcross) on seed and fruit production in highbush blueberry cv. ‘Bluecrop’ (*Vaccinium corymbosum*; Ericaceae). Am. J. Bot. 87:1584–1591. https://doi.org/10.2307/2656734 11080108

[CIT0021] Dornhaus A , Chittka L. 2004. Why do honey bees dance? Behav. Ecol. Sociobiol. 55:395–401. https://doi.org/10.1007/s00265-003-0726-9

[CIT0022] Eeraerts M , Meeus I, Berge SVD, et al 2017. Landscapes with high intensive fruit cultivation reduce wild pollinator services to sweet cherry. Agric. Ecosyst. Environ. 239:342–348. https://doi.org/10.1016/j.agee.2017.01.031

[CIT0023] Eeraerts M , DeVetter LW, Batáry P, et al 2023a. Synthesis of highbush blueberry pollination research reveals region‐specific differences in the contributions of honeybees and wild bees. J. Appl. Ecol. 60:2528–2539. https://doi.org/10.1111/1365-2664.14516

[CIT0024] Eeraerts M , Rogers E, Gillespie B, et al 2023b. Landscape-level honey bee hive density, instead of field-level hive density, enhances honey bee visitation in blueberry. Landsc. Ecol. 38:583–595. https://doi.org/10.1007/s10980-022-01562-1

[CIT0025] Eeraerts M , Chabert S, DeVetter LW, et al 2024. Pollination deficits and their relation with insect pollinator visitation are cultivar-dependent in an entomophilous crop. Agric. Ecosyst. Environ. 369:109036. https://doi.org/10.1016/j.agee.2024.109036

[CIT0026] Ehlenfeldt MK. 2001. Self- and cross-fertility in recently released highbush blueberry cultivars. HortScience 36:133–135. https://doi.org/10.21273/hortsci.36.1.133

[CIT0027] Estravis-Barcala MC , Palottini F, Macri I, et al 2021. Managed honeybees and South American bumblebees exhibit complementary foraging patterns in highbush blueberry. Sci. Rep. 11:8187. https://doi.org/10.1038/s41598-021-87729-3 33854164 PMC8046787

[CIT0028] Free JB. 1958. The drifting of honey-bees. J. Agric. Sci. 51:294–306. https://doi.org/10.1017/s0021859600035103

[CIT0029] Garratt MP , Groot GA de, Albrecht M, et al 2021. Opportunities to reduce pollination deficits and address production shortfalls in an important insect‐pollinated crop. Ecol. Appl. 31:e02445. https://doi.org/10.1002/eap.2445 34448315 PMC11475340

[CIT0030] Gibbs J , Elle E, Bobiwash K, et al 2016. Contrasting pollinators and pollination in native and non-native regions of highbush blueberry production. PLoS One 11:e0158937. https://doi.org/10.1371/journal.pone.0158937 27391969 PMC4938509

[CIT0031] Grant KJ , DeVetter LW, Melathopoulos A. 2021. Honey bee (*Apis mellifera*) colony strength and its effects on pollination and yield in highbush blueberries (*Vaccinium corymbosum*). PeerJ 9:e11634. https://doi.org/10.7717/peerj.11634 34395063 PMC8323595

[CIT0032] Guerra ME , Rodrigo J. 2015. Japanese plum pollination: a review. Sci. Horticult. 197:674–686. https://doi.org/10.1016/j.scienta.2015.10.032

[CIT0033] Hanson, E, Kelsey L. Gibberellin use for fruit set on blueberries. East Lansing (MI, USA): Michigan State University Extension; 2019. Available from: https://www.canr.msu.edu/news/gibberellin-use-for-fruit-set-on-blueberries

[CIT0034] Hoffman GD , Lande C, Rao S. 2018. A novel pollen transfer mechanism by honey bee foragers on highbush blueberry (Ericales: Ericaceae). Environ. Entomol. 47:1465–1470. https://doi.org/10.1093/ee/nvy162 30452583

[CIT0035] Isaacs R , Kirk AK. 2010. Pollination services provided to small and large highbush blueberry fields by wild and managed bees. J. Appl. Ecol. 47:841–849. https://doi.org/10.1111/j.1365-2664.2010.01823.x

[CIT0036] Isaacs R , Williams N, Ellis J, et al 2017. Integrated crop pollination: combining strategies to ensure stable and sustainable yields of pollination-dependent crops. Basic Appl. Ecol. 22:44–60. https://doi.org/10.1016/j.baae.2017.07.003

[CIT0037] Kendall LK , Gagic V, Evans LJ, et al 2020. Self‐compatible blueberry cultivars require fewer floral visits to maximize fruit production than a partially self‐incompatible cultivar. J. Appl. Ecol. 57:2454–2462. https://doi.org/10.1111/1365-2664.13751

[CIT0038] Kumarihami HMPC , Park HG, Kim SM, et al 2021. Flower and leaf bud density manipulation affects fruit set, leaf-to-fruit ratio, and yield in southern highbush ‘Misty’ blueberry. Sci. Horticult. 290:110530. https://doi.org/10.1016/j.scienta.2021.110530

[CIT0039] Li J , Broussard M, Tomer N, et al 2022. Honey bee (*Apis mellifera*) hive placement is more influential than orchard layout on the fruit set of a dioecious crop. Ecol. Model. 472:110074. https://doi.org/10.1016/j.ecolmodel.2022.110074

[CIT0040] Mallinger RE , Gaines-Day HR, Gratton C. 2017. Do managed bees have negative effects on wild bees?: a systematic review of the literature. PLoS One 12:e0189268. https://doi.org/10.1371/journal.pone.0189268 29220412 PMC5722319

[CIT0041] Mallinger R , Ternest JJ, Naranjo SM. 2021. Blueberry yields increase with bee visitation rates, but bee visitation rates are not consistently predicted by colony stocking densities. J. Econ. Entomol. 114:1441–1451. https://doi.org/10.1093/jee/toab111 34106276

[CIT0042] Melathopoulos AP , Tyedmers P, Cutler GC. 2014. Contextualising pollination benefits: effect of insecticide and fungicide use on fruit set and weight from bee pollination in lowbush blueberry. Ann. Appl. Biol. 165:387–394. https://doi.org/10.1111/aab.12143

[CIT0044] Osterman J , Theodorou P, Radzevičiūtė R, et al 2021. Apple pollination is ensured by wild bees when honey bees are drawn away from orchards by a mass co-flowering crop, oilseed rape. Agric. Ecosyst. Environ. 315:107383. https://doi.org/10.1016/j.agee.2021.107383

[CIT0045] Osterman, J, Benton F, Hellström S, Luderer‐Pflimpfl M, Pöpel‐Eisenbrandt AK, Wild BS, Theodorou P, Ulbricht C, Paxton RJ. 2023. Mason bees and honey bees synergistically enhance fruit set in sweet cherry orchards. Ecol. Evol. 13:e10289. https://doi.org/10.1002/ece3.10289 37435028 PMC10329911

[CIT0046] Pascual Tudanca MP , Debandi H, Vázquez DP. 2024. Managed honeybee hives negatively affect the reproduction of native plants in a dryland nature reserve. J. Appl. Ecol. 61:1301–1311. https://doi.org/10.1111/1365-2664.14646

[CIT0047] Peterson JD , Reiners S, Nault BA. 2013. Pollination services provided by bees in pumpkin fields supplemented with either *Apis mellifera* or *Bombus* impatiens or not supplemented. PLoS One 8:e69819. https://doi.org/10.1371/journal.pone.0069819 23894544 PMC3722171

[CIT0048] Pinheiro, J, Bates D. 2019. nlme: linear and nonlinear mixed effects models. R package version 3.1–143. Retrieved on 30 December 2019 from: https://cran.r-project.org/web/packages/nlme/nlme.pdf.

[CIT0049] R Core Team. R: A language and environment for statistical computing. Vienna, Austria: R Foundation for Statistical Computing; 2021. https://www.R-project.org/.

[CIT0050] Ramírez-Mejía AF , Lomáscolo S, Blendinger PG. 2023. Hummingbirds, honeybees, and wild insect pollinators affect yield and berry quality of blueberries depending on cultivar and farm’s spatial context. Agric. Ecosyst. Environ. 342:108229. https://doi.org/10.1016/j.agee.2022.108229

[CIT0051] Ramírez-Mejía AF , Chacoff NP, Cavigliasso P, et al 2024a. How much is enough? optimizing beehive stocking densities to maximize the production of a pollinator-dependent crop. Ecol. Model. 498:110891. https://doi.org/10.1016/j.ecolmodel.2024.110891

[CIT0052] Ramírez-Mejía AF , Chacoff NP, Lomáscolo SB, et al 2024b. Optimal pollination thresholds to maximize blueberry production. Agric. Ecosyst. Environ. 365:108903. https://doi.org/10.1016/j.agee.2024.108903

[CIT0053] Reilly JR , Artz DR, Biddinger D, et al 2020. Crop production in the USA is frequently limited by a lack of pollinators. Proc. Biol. Sci. 287:20200922. https://doi.org/10.1098/rspb.2020.0922 33043867 PMC7423660

[CIT0054] Retamales JB , Mena C, Lobos G, et al 2015. A regression analysis on factors affecting yield of highbush blueberries. Sci. Horticult. 186:7–14. https://doi.org/10.1016/j.scienta.2015.02.003

[CIT0055] Rogers SR , Tarpy DR, Burrack HJ. 2013. Multiple criteria for evaluating pollinator performance in highbush blueberry (Ericales: Ericaceae) agroecosystems. Environ. Entomol. 42:1201–1209. https://doi.org/10.1603/EN12303 24280253

[CIT0056] Santibañez F , Joseph J, Abramson G, et al 2022. Designing crop pollination services: a spatially explicit agent-based model for real agricultural landscapes. Ecol. Model. 472:110094. https://doi.org/10.1016/j.ecolmodel.2022.110094

[CIT0057] Šekulja D , Pechhacker H, Licek E. 2014. Drifting behavior of honey bees (*Apis Mellifera Carnica* Pollman, 1879) in the epidemiology of American foulbrood. Zbornik Veleučilišta u Rijeci. 2:345–358.

[CIT0058] Steffan-Dewenter I , Kuhn A. 2003. Honeybee foraging in differentially structured landscapes. Proc. Biol. Sci. 270:569–575. https://doi.org/10.1098/rspb.2002.2292 12769455 PMC1691282

[CIT0059] Strik BC , Vance AJ. 2019. Highbush blueberry cultivars differ in the relationship between seed number and berry weight during the harvest season. HortScience 54:1728–1736. https://doi.org/10.21273/hortsci14198-19

[CIT0060] Strik, BC, Finn CE, Moore PP. Blueberry cultivars for the Pacific Northwest. Pacific Northwest Publication 656; 2014. Available from: https://extension.oregonstate.edu/catalog/pub/pnw-656-blueberry-cultivars-pacific-northwest.

[CIT0061] Sun Q , Zhao X, Wu L, et al 2021. Differences in pollination efficiency among three bee species in a greenhouse and their effects on yield and fruit quality of northern highbush ‘Bluecrop’ blueberry. HortScience 56:603–607. https://doi.org/10.21273/hortsci15714-21

[CIT0062] Tamburini G , Bommarco R, Kleijn D, et al 2019. Pollination contribution to crop yield is often context-dependent: a review of experimental evidence. J. Agric. Environ. 280:16–23. https://doi.org/10.1016/j.agee.2019.04.022

[CIT0063] USDA NASS. 2024. Noncitrus fruits and nuts 2023 summary. https://usda.library.cornell.edu/concern/publications/zs25x846c.

[CIT0064] Vaissière, BE, Freitas BM, Gemmill-Herren B. Protocol to detect and assess pollination deficits in crops: a handbook for its use. Rome, Italy: Food and Agriculture Organization; 2011.

[CIT0065] Visscher PK , Seeley TD. 1982. Foraging strategy of honeybee colonies in a temperate deciduous forest. Ecology 63:1790–1801. https://doi.org/10.2307/1940121

[CIT0066] von Frisch KV. The dance language and orientation of bees. Cambridge (MA, USA): Harvard University Press; 1993.

