# Supplementary material for: Strategic honey bee hive placement improves honey bee visitation but not pollination in northern highbush blueberry
Source: J Econ Entomol. 2024 Nov 9;118(1):282–90. doi: 10.1093/jee/toae267 (PMC11818375; doi:10.1093/jee/toae267)
Supplement: toae267_suppl_Supplementary_Figures_S1-S2_Tables_S1-S4 [file toae267_suppl_supplementary_figures_s1-s2_tables_s1-s4.docx]

**Supplementary material**

Strategic Honey Bee Hive Placement Improves Honey Bee Visitation but not Pollination in Northern Highbush Blueberry

Kayla Brouwer, Maxime Eeraerts, Emma Rogers, Lauren Goldstein, Jacquelyn A. Perkins, Meghan O. Milbrath, Andony Melathopoulos, Jason Meyer, Clark Kogan, Rufus Isaacs, and Lisa Wasko DeVetter*

*Contact: [lisa.devetter@wsu.edu](mailto:lisa.devetter@wsu.edu)

A


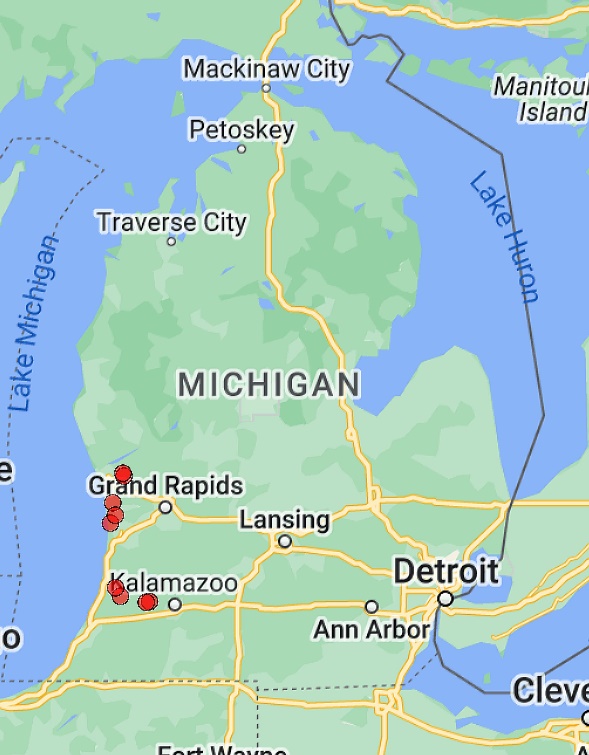


B


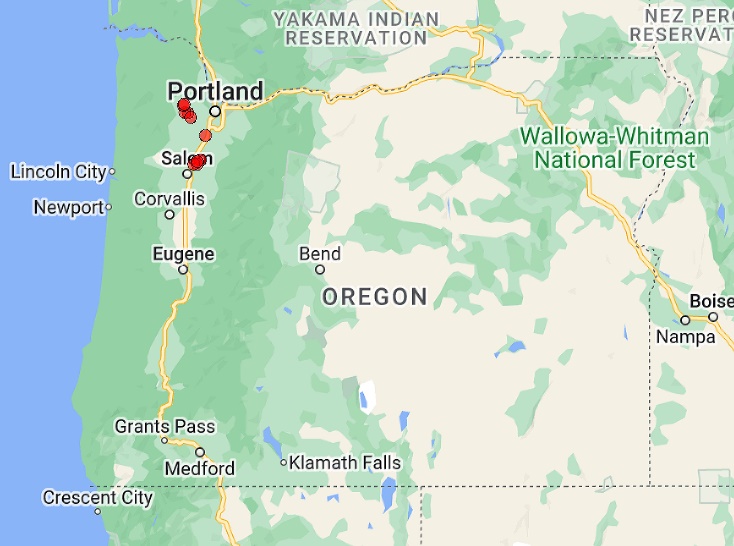


C


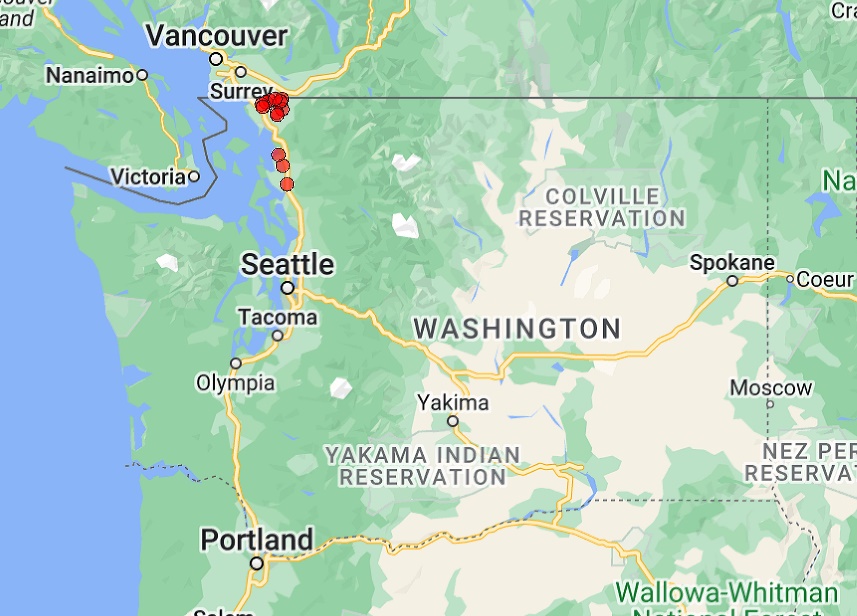


**Fig. S1.** Map of the study locations in Michigan (A), Oregon (B), and Washington (C), USA. For this study Michigan is considered as the Midwest region, whereas Oregon and Washington are considered as the Pacific Northwest. Red dots indicate the location of blueberry fields sampled in 2021 and 2022.

**Table S1.** Overview of all sampled fields per region. For every field the state, field ID, year(s) of sampling, placement treatment, field size (hectare), and hive density (per hectare) are provided.

| **Region** | **State** | **Field ID** | **Year(s) of sampling** | **Placement treatment** | **Field size** | **Hive density in 2021** | **Hive density in 2022** |
| --- | --- | --- | --- | --- | --- | --- | --- |
| Midwest | MI | M01 | 2021 and 2022 | Dispersed | 6.5 | 3.50 | 3.91 |
| Midwest | MI | M02 | 2021 | Dispersed | 6.8 | 1.80 | NA |
| Midwest | MI | M03 | 2021 and 2022 | Dispersed | 2.5 | 4.00 | 2.29 |
| Midwest | MI | M04 | 2021 and 2022 | Clumped | 1.5 | 2.00 | 1.83 |
| Midwest | MI | M05 | 2021 and 2022 | Dispersed | 1.4 | 2.00 | 2.57 |
| Midwest | MI | M06 | 2021 and 2022 | Clumped | 2.5 | 4.00 | 3.27 |
| Midwest | MI | M07 | 2021 and 2022 | Clumped | 2.1 | 3.50 | 2.70 |
| Midwest | MI | M08 | 2021 and 2022 | Clumped | 3.3 | 2.80 | 2.19 |
| Midwest | MI | M09 | 2022 | Dispersed | 5.7 | NA | 5.09 |
| PNW | OR | PNW01 | 2021 and 2022 | Clumped | 4.1 | 4.00 | 1.66 |
| PNW | OR | PNW02 | 2021 | Clumped | 3.9 | 4.00 | NA |
| PNW | OR | PNW03 | 2021 | Dispersed | 2.0 | 4.00 | NA |
| PNW | OR | PNW04 | 2021 | Dispersed | 1.9 | 4.00 | NA |
| PNW | OR | PNW05 | 2021 and 2022 | Clumped | 3.5 | 4.00 | 3.72 |
| PNW | OR | PNW06 | 2021 and 2022 | 2021: Dispersed, 2022: Clumped | 1.5 | 4.00 | 4.67 |
| PNW | OR | PNW07 | 2022 | Dispersed | 5.7 | NA | 4.56 |
| PNW | OR | PNW08 | 2022 | Clumped | 7.6 | NA | 4.66 |
| PNW | WA | PNW09 | 2021 and 2022 | Dispersed | 8.1 | 6.72 | 5.75 |
| PNW | WA | PNW10 | 2021 and 2022 | Clumped | 6.8 | 6.14 | 5.11 |
| PNW | WA | PNW11 | 2021 and 2022 | Clumped | 7.7 | 6.24 | 3.50 |
| PNW | WA | PNW12 | 2021 and 2022 | Dispersed | 5.2 | 3.30 | 6.73 |
| PNW | WA | PNW13 | 2021 | Dispersed | 6.3 | 1.43 | NA |
| PNW | WA | PNW14 | 2021 and 2022 | Clumped | 5.3 | 6.99 | 4.09 |
| PNW | WA | PNW15 | 2021 and 2022 | Clumped | 7.2 | 3.24 | 2.81 |
| PNW | WA | PNW16 | 2021 and 2022 | Dispersed | 6.0 | 7.25 | 5.21 |
| PNW | WA | PNW17 | 2021 and 2022 | 2021: Dispersed, 2022: Clumped | 7.1 | 4.17 | 6.10 |
| PNW | WA | PNW18 | 2021 and 2022 | Clumped | 7.8 | 4.58 | 4.58 |
| PNW | WA | PNW19 | 2021 and 2022 | Clumped | 8.4 | 9.35 | 8.45 |
| PNW | WA | PNW20 | 2021 and 2022 | Dispersed | 7.9 | 3.06 | 3.49 |
| PNW | WA | PNW21 | 2022 | Dispersed | 4.8 | NA | 1.36 |

**Table S2.** Correlation between hive placement treatment and field-level hive density. By means of linear mixed-effects model we tested if field-level hive density varied between hive placement treatment. Fixed variables in this model included hive placement treatment, region, and their interaction with Year nested in Field_ID as random variables.

| **Response** | **Fixed factors** | **F** | **P** |
| --- | --- | --- | --- |
| Field-level hive density | Honey bee hive placement treatment | 1.51 | 0.23 |
|  | Region | 6.07 | 0.02 |
|  | Honey bee hive placement treatment: Region | 1.17 | 0.29 |


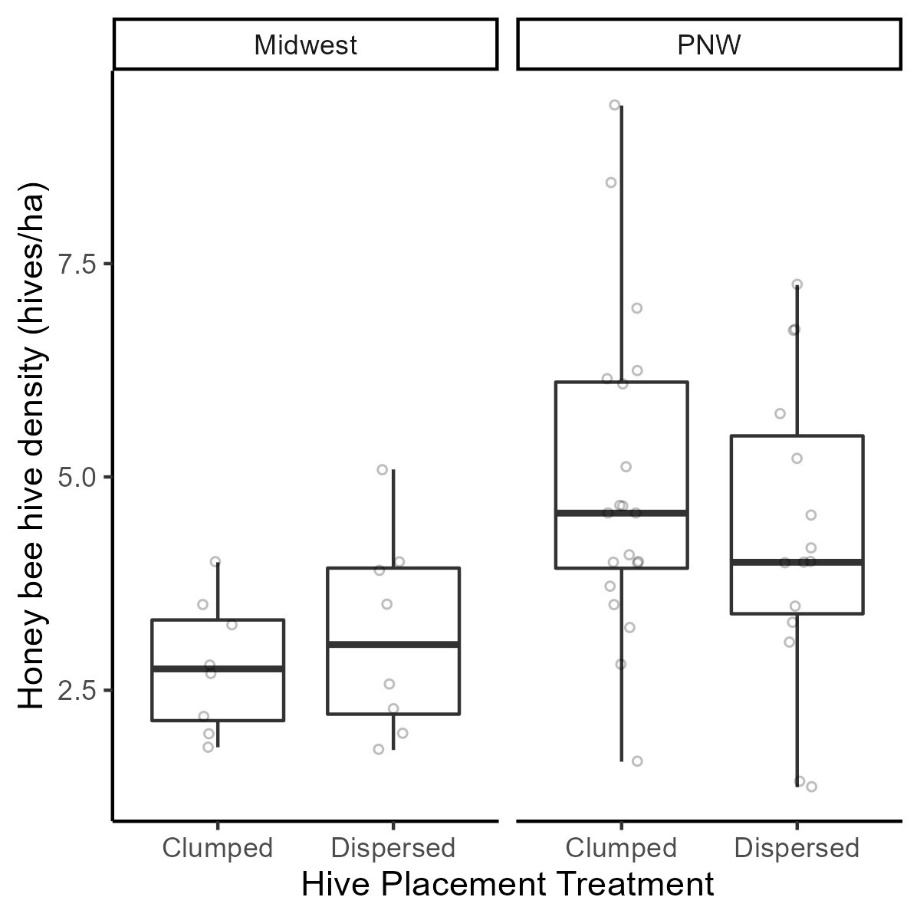


**Fig. S2.** Honey bee hive density in relation to hive placement treatment for both study regions.

**Table S3.** Results of Pearson correlation tests between honey bee visitation and temperature in the different regions and years.

| **Region** | **Year** | **Corr. coef.** | **P** |
| --- | --- | --- | --- |
| Midwest | 2021 | 0.21 | <0.01 |
|  | 2022 | -0.29 | <0.01 |
| PNW | 2021 | 0.35 | <0.001 |
|  | 2022 | 0.29 | <0.001 |

**Table S4.** Result of linear mixed-effect models excluding outliers, assessing the effect of honey bee hive placement, hive density (per hectare), and region on fruit set and weight per 100 berries in highbush blueberry. Full models are reported with their F-statistic, P-values, and marginal R².

| **Response variable** | **Full model** | **R²m** | **Fixed variables** | **F** | **P** |
| --- | --- | --- | --- | --- | --- |
| Fruit set | Region*Placement + Region*Density | 0.29 | Region | 2.09 | 0.16 |
|  |  |  | Placement | 0.01 | 0.94 |
|  |  |  | Density | 0.42 | 0.53 |
|  |  |  | Region:Placement | 0.20 | 0.66 |
|  |  |  | Region:Density | 0.02 | 0.89 |
| Weight per 100 berries | Region*Placement + Region*Density | 0.10 | Region | 0.17 | 0.68 |
|  |  |  | Placement | 0.23 | 0.64 |
|  |  |  | Density | 0.07 | 0.80 |
|  |  |  | Region:Placement | 0.76 | 0.40 |
|  |  |  | Region:Density | 0.00 | 0.98 |
